# Safety and Efficacy of Post-Dilation in Percutaneous Coronary Intervention Using Polymer-Free Ultrathin Strut Sirolimus-Probucol Coated Drug-Eluting Stents

**DOI:** 10.3390/medicina59091649

**Published:** 2023-09-12

**Authors:** Yonghoon Shin, Yoonsun Won, Taeil Yang, Joohan Kim, Joonpyo Lee, Jeongduk Seo, Albert Youngwoo Jang, Minsu Kim, Pyung Chun Oh, Kyounghoon Lee, Woong Chol Kang, Seung Hwan Han, Soon Yong Suh

**Affiliations:** 1 Department of Critical Care Medicine, Samsung Medical Center, Sungkyunkwan University School of Medicine, Seoul 06351, Republic of Korea; fibrillary@gmail.com; 2 Department of Cardiology, Gil Medical Center, Gachon University College of Medicine, Incheon 21565, Republic of Korea; 3 Department of Internal Medicine, Cardiovascular Center, Chinjujeil Hospital, Jinju 52709, Republic of Korea

**Keywords:** percutaneous coronary intervention, post-dilation, polymer-free ultrathin strut sirolimus- and probucol-eluting stents

## Abstract

*Background and Objectives*: Polymer-free ultrathin strut sirolimus- and probucol-eluting stents (PF-SES) are recognized as safe and effective in diverse patient populations, although the implications of post-dilation during stent implantation remain underexamined. *Materials and Methods*: In this study, patients implanted with PF-SES at Gachon University Gil Medical Center between December 2014 and February 2018 were evaluated. Major adverse cardiovascular events (MACE), encompassing nonfatal myocardial infarction (MI), nonfatal stroke, and cardiovascular death were identified as primary outcomes, with secondary outcomes including target vessel revascularization (TVR), target lesion revascularization (TLR), and in-stent restenosis (ISR). *Results*: Of the 384 initial patients, 299 were considered eligible for analysis. The groups, delineated by those undergoing post-dilation (143 patients) and those not (156 patients), exhibited comparable rates of primary outcomes [hazard ratio (HR), 2.17; 95% confidence interval (CI), 0.40 to 11.87; *p* = 0.37]. The outcomes remained consistent irrespective of the post-dilation status and were similarly unaffected in multivariate analyses (HR, 2.90; 95% CI, 0.52 to 16.34; *p* = 0.227). *Conclusions*: These results suggest that the clinical outcomes of patients with post-dilation were similar to that of those without post-dilation in those with the polymer-free sirolimus- and probucol-eluting stents.

## 1. Introduction

Coronary artery disease poses a substantial global health challenge, significantly impacting morbidity and mortality worldwide [1]. With the progression of medical technology, percutaneous coronary intervention (PCI) has emerged as a fundamental element in the treatment of coronary artery disease, with stent implantation steadily becoming the centerpiece of this intervention [2,3]. Initially, PCI involved the use of bare-metal stents, essentially serving as metal frameworks. However, due to the high propensity for restenosis brought about by neointimal hyperplasia, these stents have evolved into newer generations of drug-eluting stents. These advanced stents are coated with drugs designed to prevent excessive overgrowth [4]. However, stent underexpansion, which is the result of insufficient lumen dimensions following the procedure, can occur with any stent type and may lead to restenosis, target vessel revascularization (TVR), and stent thrombosis stents [5,6,7,8,9].

Post-dilation has been proposed as a strategy to address this problem of stent underexpansion [10]. Post-dilation aims to optimize stent expansion and reduce the likelihood of adverse clinical outcomes by further expanding the stent after stent implantation [11,12,13,14]. Several large studies have demonstrated that improved post-dilatation stent deployment is associated with better clinical outcomes, and these findings underscore the importance of achieving stent optimization by ensuring that the minimum stent area is sufficiently covered with intracoronary imaging guidance [10,15,16,17]. Still, the application of post-dilation procedures is not without dispute, raising worries that they may induce extra harm to the coronary arteries and promote intimal hyperplasia [18,19]. Moreover, failure of stent optimization caused by over-expansion after post-dilatation may lead to an increased risk of major adverse cardiovascular events (MACE) and stent fractures [20,21,22,23].

Delayed healing might transpire due to hypersensitivity reactions elicited by the stent polymer, potentially linked to an elevated risk of stent thrombosis [24]. Alternatively, the thickness of the stent strut may provoke a localized inflammatory response at the lesion within the insertion site, playing a critical role in determining strut coverage [25,26]. To address these conventional drug eluting stent limitations, stent technology has persistently evolved, resulting in the development of polymer-free ultrathin strut sirolimus- and probucol-eluting stents (PF-SES) (Coroflex^®^ ISAR, B. Braun Melsungen, Germany) [27]. Probucol is a liposoluble antioxidant that prevents low-density lipoprotein oxidation and inhibits inflammatory cell adherence to the vascular endothelium, limiting macrophage accumulation and free radical production, and was effective in reducing restenosis in both animal models and clinical trials [28,29,30,31]. Probucol’s inherent lipophilicity, when utilized as part of a stent coating matrix, may have the ability to decelerate the release of sirolimus, enhancing its therapeutic potential against restenosis [32,33]. Polymer-free ultrathin-strut stents aim to mitigate local inflammation and enhance strut coverage by removing the polymer element and minimizing strut thickness, thereby reducing complication risks. Several studies have corroborated these assertions, with various reports indicating lower incidences of stent thrombosis, target lesion revascularization (TLR), and MACE following PF-SES implantation [27,34,35,36,37,38].

However, like all medical advancements, PF-SES has its own limitations. Concerns have been raised about the radial strength of ultrathin strut stents, particularly in the context of lesions that demand increased radial force, such as coronary chronic total occlusions (CTOs). In such scenarios, ultrathin strut stents have been observed to demonstrate higher stent recoil, late lumen loss, and restenosis compared to traditional stents [39,40]. Despite these reservations, the effect of post-dilation on PF-SES remains under-researched. While post-dilation is a frequently employed strategy for under-expanded stents, its implications on PF-SES are still not entirely understood, making this a crucial topic to explore. Consequently, this study was undertaken as an in vivo study to assess the long-term clinical outcomes and safety of post-dilation in patients undergoing PCI with PF-SES rather than an in vitro analysis.

## 2. Materials and Methods

### 2.1. Study Design and Population

This retrospective, observational, single-center study included patients who underwent PCI with PF-SES (Coroflex^®^ ISAR, B. Braun Melsungen, Germany) implantation at Gachon University Gil Medical Center between December 2014 and February 2018. The criteria for PCI encompassed significant de novo coronary lesions (over 70% stenosis identified through coronary angiography) as well as documented myocardial ischemia. This evidence of ischemia was derived not solely from the patient’s typical angina, indicative of ischemic heart disease, but also from positive outcomes on exercise stress or imaging tests that necessitated PCI. The range of conditions necessitating PCI extended from chronic coronary syndromes (CCS) to acute coronary syndromes (ACS), such as unstable angina and myocardial infarction. The study population was divided into two groups: those who underwent post-dilation (post-dilation group) and those who did not (no post-dilation group). The analysis excluded patients who had received different types of implanted stents, multiple PF-SESs, or a history of coronary artery bypass grafting. The current study conformed to the Declaration of Helsinki (sixth revision). The Institutional Review Board approved this study (GDIRB 2020-132, 7 April 2020) and waived the requirement for informed consent due to the retrospective observational nature of the study.

### 2.2. Stent Implantation and Post-Dilation Procedure

All the patients included in the study underwent stent implantation with PF-SES. In the framework of the polymer-free stent, there is a pre-assembled fine cobalt–chromium strut (50/60 µm in size). Sirolimus serves as an effective anti-proliferative agent, while probucol acts as a controlling agent for the drug’s release [27]. Before the procedure, all the patients scheduled for PCI who were not usually on antiplatelet therapy were administered a loading dose of antiplatelet agents. The use of either femoral or radial vascular access was allowed, with the suggested introducer sheaths being no less than 5 Fr in diameter. To mitigate the risk of thrombosis, a pre-emptive bolus of 5000 units of unfractionated heparin (UFH) was administered intravenously prior to the coronary angiography. Following sheath insertion at the start of the procedure, an additional UFH dosage of 50 U/kg was given, and further doses were administered as necessary throughout the intervention to maintain an activated clotting time within the range of 250 to 300 s. In every patient, the reference vessel diameter and minimal lumen diameter were meticulously documented. The implantation procedure was carried out following standard clinical practices, including pre-dilation if deemed necessary. Immediately following the stent deployment, stent expansion was assessed using both the enhanced stent visualization (ESV) with CLEARstent technology (Siemens Healthcare, Munich, Germany) or intravascular ultrasonography, with the ESV being the predominant evaluation method in most cases [41]. To use the ESV technique, approximately 30 frames of digital cine (over 3 s) were acquired without contrast medium with the guide catheter and balloon markers within the stent segment in view. Post-dilation was performed if stent expansion was inadequate. Sufficient stent expansion using the ESV technique was defined by the absence of any signs of focal under-expansion. This could be identified by a lack of protrusion of the stent strut, visual continuity of the stent struts, and the stent’s minimum diameter being greater than 80% of the reference diameter. In addition, an adequate stent area was considered to be more than 80% of the mean reference lumen area or an absolute minimum stent area greater than 5.5 mm^2^ when assessed using intravascular ultrasonography for stenosis not located in the left main coronary artery [42]. However, for stenosis in the left main coronary artery, an absolute minimum stent area of more than 7 mm^2^ for the distal segment and more than 8 mm^2^ for the proximal segment were required. The decision to perform post-dilation was made by the treating physician based on angiographic imaging using ESV or based on intravascular imaging findings. When performed, post-dilation was conducted using either a non-compliant balloon with a diameter equal to or 0.5 mm larger than the nominal stent diameter or a semi-compliant balloon inflated to a high pressure (≥ 16 atm) with a supranominal diameter.

### 2.3. Data Collection and Follow-Up

The baseline demographics, clinical characteristics, angiographic and procedural data, and post-procedural medications were extracted from the electronic medical records. The baseline demographics accounted for factors such as age and gender, as well as lifestyle-related information like smoking. Meanwhile, the clinical characteristics encompassed the patient’s past medical history; family history of coronary artery disease; presence or absence of risk factors for coronary artery disease such as hypertension, diabetes mellitus, dyslipidemia, or chronic kidney disease; and any previous history of myocardial infarction, heart failure, or stroke. Regarding the angiographic data, variables like the degree of coronary artery stenosis, location and length of the lesion, and presence of multi-vessel disease were meticulously recorded. The procedural data included the type of stents used, whether balloon pre-dilation or post-dilation was performed, the maximum pressure applied, and the final result of the procedure in terms of residual stenosis and thrombolysis in myocardial infarction flow grade. For the follow-up phase, the patients were asked to return for regular outpatient visits, which provided an opportunity to assess their clinical status, medication compliance, and the occurrence of any potential adverse events. Routine clinical follow-ups were scheduled at specific intervals—after 6 months, 1 year, 2 years, and 3 years—following the index PCI. Additional angiographic follow-ups were conducted as necessary, such as in the presence of ischemic symptoms, to meticulously observe stent patency and identify any cases of in-stent restenosis or other complications.

### 2.4. Clinical Outcomes

This study was an in vivo investigation concentrating on clinical outcomes rather than a bench test examining the deformation of the stent platform following post-dilation. The primary outcome was MACE, a composite of nonfatal myocardial infarction, nonfatal stroke, and cardiovascular death. The secondary outcomes included TVR, TLR, and ISR. Myocardial infarction was defined according to the third universal definition of myocardial infarction. Stroke was defined as an acute neurological deficit lasting more than 24 h with evidence of brain infarction in imaging. TVR was defined as any repeat PCI in the target vessel. TLR was defined as repeat PCI within the index procedure stent or 5 mm edge. ISR was defined as a diameter stenosis of ≥50% within the stent or 5 mm proximal or distal to the stent, assessed either using angiography or intravascular imaging.

### 2.5. Statistical Analysis

Continuous variables were presented as means ± standard deviations and compared using a Student’s *t*-test. Categorical variables were presented as frequencies and percentages and compared using a Chi-square test. Hazard ratios (HR) with 95% confidence intervals (CI) were calculated using Cox proportional hazards regression models to assess the impact of post-dilation on the primary and secondary outcomes. Multivariate analyses were performed to adjust for potential confounding factors, including hypertension, diabetes mellitus, B2 or C lesion, and calcification. Kaplan–Meier curves were used to visualize the cumulative event rates over time for the primary and secondary outcomes. A two-sided *p*-value < 0.05 was considered statistically significant. All the analyses were performed using SPSS software version 29 (IBM Corp., Armonk, NY, USA).

## 3. Results

### 3.1. Patient Characteristics and Procedural Data

**Figure 1:**
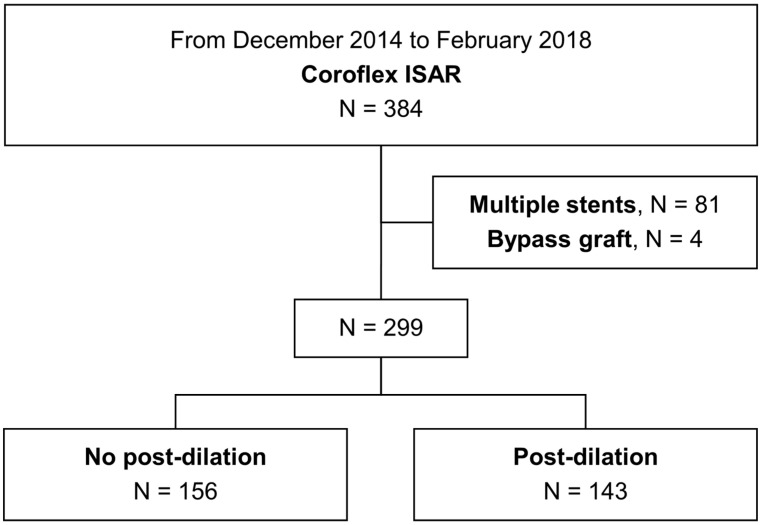
Flow diagram of the separation into groups.

A total of 384 patients who underwent PCI with PF-SES implantation were initially identified. The final study population consisted of 299 patients after excluding 85 patients who had different types of stents implanted, those who had multiple PF-SESs implanted, or those who had a history of bypass grafting. Among them, 156 patients did not undergo post-dilation (no post-dilation group) and 143 patients underwent post-dilation (post-dilation group) (Figure 1). The baseline demographics and clinical characteristics were largely comparable between the two groups, with the exception of chronic kidney disease, which was more common in the no post-dilation group (9.0% vs. 2.8%, *p* = 0.025) (Table 1).

In terms of coronary lesion characteristics, the type and location of treated vessels did not differ between the two groups, but the post-dilation group had more complex lesions. Specifically, B2 or C lesions and calcific lesions were more common in the post-dilation group compared to the no post-dilation group (26.6% vs. 53.6%, *p* < 0.001; 5.1% vs. 23.8%, *p* < 0.001). While there was no significant difference in the stent diameter between the two groups, the stent length was longer in the post-dilation group. All the lesions were evaluated for the appropriateness of stent expansion using intravascular ultrasonography or ESV, with the majority of cases utilizing ESV. The medications prescribed at discharge were not significantly different between the two groups (Table 1).

### 3.2. Primary Outcomes

**Figure 2:**
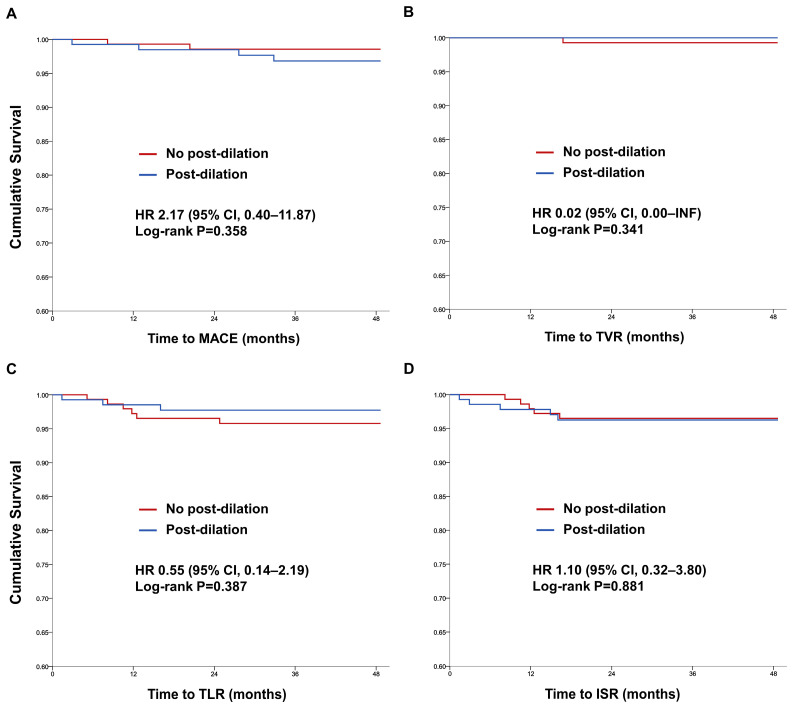
Kaplan–Meier curves of clinical outcomes. (**A**) MACE, (**B**) TVR, (**C**) TLR, (**D**) ISR. ISR: in-stent restenosis; MACE: major adverse cardiovascular events; TLR: target lesion revascularization; TVR: target vessel revascularization.

During the follow-up period, the primary outcome of MACE occurred in two patients (1.3%) in the no post-dilation group and four patients (2.8%) in the post-dilation group. The hazard ratio for MACE was 2.17 (95% confidence interval, 0.40 to 11.87; *p* = 0.358), indicating no statistically significant difference between the two groups (Figure 2A and Table 2).

### 3.3. Secondary Outcomes

Myocardial infarction was observed in one patient (0.6%) in the no post-dilation group and two patients (1.4%) in the post-dilation group, with a hazard ratio of 2.18 (95% confidence interval, 0.20 to 24.03; *p* = 0.525). TVR was observed in one patient (0.6%) in the no post-dilation group and no patients (0.0%) in the post-dilation group, with a hazard ratio of 0.02 (95% confidence interval, 0.00 to INF; *p* = 0.619). TLR occurred in six patients (3.8%) in the no post-dilation group and three patients (2.1%) in the post-dilation group, with a hazard ratio of 0.55 (95% confidence interval, 0.14 to 2.19; *p* = 0.394). ISR was observed in five patients (3.2%) in the no post-dilation group and five patients (3.5%) in the post-dilation group, with a hazard ratio of 1.10 (95% confidence interval, 0.32 to 3.80; *p* = 0.881). All the secondary outcomes were comparable between the two groups, with no statistically significant differences detected (Figure 2B–D and Table 2).

### 3.4. Multivariate Analysis

After adjusting for potential confounders, including hypertension, diabetes mellitus, chronic kidney disease, B2 or C lesions, and calcification, the results remained consistent with the unadjusted analysis. There were no significant differences in the primary or secondary outcomes between the no post-dilation and post-dilation groups (Table 2).

## 4. Discussion

This study evaluated the impact of post-dilation on clinical outcomes in patients undergoing PCI using PF-SES. Post-dilation can have a direct impact on the stent strut and polymer. Post-dilation can cause not only radial structural deformation of the stent strut but also longitudinal deformation, resulting in stent elongation [43]. In addition, an in vitro study analyzing the effects of intravascular lithotripsy on stent polymers showed that calcified lesions and post-dilation caused more extensive damage to the polymers than intravascular lithotripsy [44]. This study may provide valuable insights into the safety and effectiveness of post-dilation, especially in the context of ultrathin strut polymer-free stents.

The baseline clinical and angiographic characteristics of the study population suggest some important clinical implications. For instance, the proportion of ST elevation ACS was numerically lower in the post-dilation group (11% vs. 7.0%, Table 1), which may reflect a hesitancy by physicians to perform post-dilation in the setting of acute myocardial infarction due to concerns about potential harm [45]. ACS and CCS have different pathogenesis and clinical outcomes. However, we confirmed that the rates of ACS and CCS were not significantly different between the two comparison groups. Moreover, the methodology of including both ACS and CCS in the analysis without differentiating between them is in line with other studies on stent optimization [42]. We would like to see further studies that are specifically designed to compare the efficacy of post-dilation in ACS and CCS patients. Additionally, the proportion of ostial lesions was relatively higher in the post-dilation group (1.3% vs. 6.3%), indicating that the need for post-dilation might be influenced by the lesion location. 

The most significant observation was the noticeably higher proportion of complex lesions, B2 or C lesions (26.6% vs. 53.6%, *p* < 0.001), and calcified lesions (5.1% vs. 23.8%, *p* < 0.001) in the post-dilation group. Also, the stent length was significantly longer in the post-dilation group (17.67 mm vs. 18.80 mm, *p* = 0.029). As a result, the stents used in the post-dilation group may have been longer in order to cover the long lesion. This finding strongly suggests that post-dilation was more frequently employed in complex lesion scenarios. The angiographic characteristics of type B2 and C lesions, as classified by the modified American College of Cardiology (ACC) and American Heart Association (AHA) lesion classification, were regarded as complex lesions [46]. These had a high occurrence of target vessel myocardial infarction and ischemia-induced target lesion revascularization, thereby leading to a significant rate of target lesion failure [47]. Given the evidence that under-expansion tends to happen in complex lesions, resulting in undesirable outcomes such as restenosis, in the 2018 European Society of Cardiology and European Association for Cardio-Thoracic Surgery guidelines on myocardial revascularization, it is advocated that unexpanded stents should be assertively managed by high-pressure dilation using a noncompliant balloon [48]. 

The results of this study showed that post-dilation did not significantly affect the incidence of MACE, TVR, TLR, or ISR despite a higher prevalence of complex lesions (B2 or C lesions and calcifies lesions) in the post-dilation group (Figure 2 and Table 2). The primary and secondary outcomes were comparable between the post-dilation and no post-dilation groups, suggesting that post-dilation can be safely performed in complex lesions when using PF-SES.

The present study has several limitations. Firstly, given its retrospective, observational, single-center design, the scope of the results may be limited, potentially restricting their applicability in a broader context. Future prospective multicenter trials with larger sample sizes and randomized allocation to post-dilation are warranted to confirm these findings and elucidate the precise role of post-dilation in patients receiving PF-SES. We believe that our results can serve as a foundation for future studies that can provide more robust evidence for the effects of post-dilation. Second, the absence of a uniform evaluation method of stent expansion represents another limitation. The use of intravascular imaging was inconsistent and dependent on individual physician discretion, which may introduce selection bias. We acknowledge that intravascular imaging is the gold standard for evaluating stent expansion, but intravascular ultrasonography has the limitation that it is not covered by health insurance in Korea and is expensive. Also, optical coherence tomography is not available at our institution, so we could not apply it. We used ESV, a fluoroscopy-based evaluation method, as an alternative. ESV has been shown to be a reliable alternative to intravascular imaging in some studies [41,49,50,51,52,53]. In our study, all the lesions were evaluated for stent expansion using intravascular ultrasonography or ESV immediately after stent deployment at a nominal pressure during PCI. Post-dilation was performed if the stent expansion was inadequate. Therefore, we did not routinely perform post-dilation or base the decision to post-dilate on operator preference. 

Furthermore, the imbalance in lesion complexity between groups is another limitation of our study. This study did not exclusively analyze calcified lesions, which is a limitation. We adjusted for this imbalance as much as possible using a multivariate Cox regression analysis. The observation of more complex lesions in the post-dilation group may be due to the fact that post-dilation is typically performed when stent under-expansion occurs, and stent under-expansion is more likely to occur in complex lesions. We believe that our findings are still clinically meaningful, as we showed that post-dilation did not lead to worse clinical outcomes in patients with more complex lesions. However, we would like to see future studies with a more balanced design to further confirm our findings. In our study, only two left main lesions were included in the post-dilation group due to the maximum available size of PF-SES being 3.5 mm at our institution. The necessity of using different stents for larger left main lesions limits our understanding of the effects of post-dilation with PF-SES in these cases, and we acknowledge this as a constraint of our study. These limitations obscure insights into the effects of post-dilatation on PF-SES in left main lesions, suggesting that further studies in left main lesions are warranted. The last limitation of this study was the relatively small number of events. This may suggest that the PCI was carried out by experienced and skilled interventionists. We anticipate that this limitation could be addressed in larger, prospective, future studies. Despite these limitations, the study provides valuable insights into the clinical outcomes of PF-SES implantation with and without post-dilation, particularly in patients with more complex lesions. These findings have important implications for clinical practice, as they suggest that post-dilation may be safely performed when indicated, which could help to optimize stent deployment and improve patient outcomes.

## 5. Conclusions

In conclusion, this study demonstrated that the clinical outcomes were comparable between patients with and without post-dilation in PF-SES implantation, even in the presence of more complex lesions. These findings suggest that post-dilation using PF-SES may be a safe and effective method. Further research is needed to better understand the role of post-dilation and identify specific subgroups of patients who might benefit from this procedure.

## Figures and Tables

**Table 1:** Baseline clinical and angiographic characteristics.

	No Post-Dilation	Post-Dilation	*p*
Patients, *n*	156	143	
Male gender, *n* (%)	103 (66.0)	93 (65.0)	0.857
Age, years ± SD	67.30 ± 12.20	66.04 ± 11.16	0.354
Current smoker, *n* (%)	59 (37.8)	43 (30.1)	0.368
Hypertension, *n* (%)	97 (62.2)	85 (59.4)	0.628
Diabetes, *n* (%)	60 (38.5)	48 (33.6)	0.379
Dyslipidemia, *n* (%)	23 (14.7)	18 (12.6)	0.588
Stroke, n (%)	7 (4.5)	12 (8.4)	0.384
Family history of CAD, *n* (%)	6 (3.8)	7 (4.9)	0.657
Previous MI, *n* (%)	11 (7.1)	13 (9.1)	0.517
Previous PCI, *n* (%)	34 (21.8)	41 (28.7)	0.171
LVEF, % ± SD (*n*)	57.65 ± 12.88 (81)	57.46 ± 14.06 (66)	0.628
Heart failure, *n* (%)	35 (22.4)	23 (16.1)	0.165
Peripheral artery disease, *n* (%)	3 (1.9)	1 (0.7)	0.358
Chronic kindney disease, *n* (%)	14 (9.0)	4 (2.8)	0.025
Indication for procedure—*n* (%)			0.130
STE-ACS	17 (11.0)	10 (7.0)	
NSTE-ACS	109 (70.3)	115 (80.4)	
CCS	29 (18.7)	18 (12.6)	
Lesions characteristics—*n* (%)			
Vessels treated			0.188
Left main	0 (0.0)	2 (1.9)	
Left anterior descending	61 (39.1)	71 (49.7)	
Left circumflex	41 (26.3)	32 (22.4)	
Right coronary	53 (34.0)	37 (25.9)	
Lesion location			0.218
Ostial location	2 (1.3)	9 (6.3)	
Proximal location	47 (30.1)	45 (31.5)	
Mid location	80 (51.3)	70 (49.0)	
Distal location	20 (12.8)	12 (8.4)	
Lesion complexity			
B2 or C lesion	41 (26.6)	64 (53.6)	<0.001
Total occlusion	4 (2.6)	3 (2.1)	0.790
Calcification	8 (5.1)	34 (23.8)	<0.001
Bifurcation	2 (1.3)	3 (2.1)	0.583
Procedural characteristics—mm ± SD			
Reference vessel diameter	2.91 ± 0.42	2.99 ± 0.42	0.112
Minimal lumen diameter	0.29 ± 0.35	0.32 ± 0.30	0.408
Stent			
Diameter	2.87 ± 0.39	2.92 ± 0.39	0.273
Length	17.67 ± 4.68	18.80 ± 4.13	0.029
ESV, *n* (%)	149 (95.5)	124 (86.7)	0.007
IVUS, *n* (%)	7 (4.5)	19 (13.3)	0.007
Medication at discharge—*n* (%)			
Aspirin	156 (100)	140 (97.9)	0.069
P2Y_12_ inhibitor	156 (100)	141 (98.6)	0.138
Clopidogrel	116 (74.4)	99 (69.2)	0.324
Ticagrelor	44 (28.2)	43 (30.1)	0.723
Prasugrel	3 (1.9)	1 (0.7)	0.358
Oral anticoagulant	6 (3.8)	8 (5.6)	0.475
Statin	134 (85.9)	112 (85.3)	0.886
Beta-blocker	96 (61.5)	85 (59.4)	0.711
ACE inhibitor or ARB	104 (66.7)	85 (59.4)	0.196

Values are presented as mean ± standard deviation or as number (%). ACE: angiotensin-converting enzyme; ARB: angiotensin receptor blocker; CAD: coronary artery disease; CCS: chronic coronary syndrome; ESV: enhanced stent visualization; IVUS: intravascular ultrasonography; LVEF: left ventricular ejection fraction; MI: myocardial infarction; NSTE-ACS: non-ST elevation acute coronary syndrome; PCI: percutaneous coronary intervention; STE-ACS: ST elevation acute coronary syndrome.

**Table 2:** Multivariate Cox regression analysis of clinical outcomes.

		No Post-Dilatio *n* (%)	Post-Dilatio *n* (%)	HR	95% CI	*p*
MACE	Unadjusted	2 (1.3)	4 (2.8)	2.17	0.40–11.87	0.370
	Adjusted *	-	-	2.90	0.52–16.34	0.227
MI	Unadjusted	1 (0.6)	2 (1.4)	2.18	0.20–24.03	0.525
	Adjusted	-	-	2.61	0.23–29.88	0.440
TVR	Unadjusted	1 (0.6)	0 (0.0)	0.02	0.00–INF	0.619
	Adjusted	-	-	0.04	0.00–INF	0.678
TLR	Unadjusted	6 (3.8)	3 (2.1)	0.55	0.14–2.19	0.394
	Adjusted	-	-	0.47	0.10–2.16	0.334
ISR	Unadjusted	5 (3.2)	5 (3.5)	1.10	0.32–3.80	0.881
	Adjusted	-	-	0.95	0.25–3.55	0.934

* Adjusted for hypertension, diabetes mellitus, chronic kidney disease, B2 or C lesion, and calcification. CI: confidence interval; HR: hazard ratio; ISR: in-stent restenosis; MACE: major adverse cardiovascular events; MI: myocardial infarction; TLR: target lesion revascularization; TVR: target vessel revascularization.

## Data Availability

The data collected for this study, including individual patient data, will not be made available.
